# Editorial: The Role of the Basal Ganglia in Somatosensory-Motor Interactions: Evidence From Neurophysiology and Behavior

**DOI:** 10.3389/fnhum.2019.00451

**Published:** 2020-01-08

**Authors:** Martijn Beudel, Antonella Macerollo, Matt J. N. Brown, Robert Chen

**Affiliations:** ^1^Department of Neurology, Amsterdam Neuroscience Institute, Amsterdam University Medical Centre, Amsterdam, Netherlands; ^2^School of Psychology, University of Liverpool, Liverpool, United Kingdom; ^3^Department of Kinesiology, California State University Sacramento, Sacramento, CA, United States; ^4^Division of Neurology, Department of Medicine, University of Toronto, Toronto, ON, Canada

**Keywords:** basal ganglia, deep brain stimulation, Parkinson's disease, dystonia, oscillations, somatosensory, motor

Sensory-motor interactions offer critical mechanisms for how we move. Somatosensory information, from cutaneous, muscle, joint, and tendon receptors, are known to provide the central nervous system (CNS) with information about the body and the environment (e.g., objects). In turn, somatosensory input provided to the CNS can be relayed to motor areas to assist in the preparation, execution, and correction of movements.

The basal ganglia are known to receive input from both cortical somatosensory and motor areas, but the significance and the contribution of these interactions for various aspects of human movement remains enigmatic. A variety of different neurophysiological techniques have been used to investigate the role of the basal ganglia in somatosensory-motor interactions including electroencephalography (EEG), magnetoencephalography (MEG), magnetic resonance imaging (MRI), recordings from deep brain stimulation (DBS) electrodes, and combining DBS with transcranial magnetic stimulation (TMS) over the cortex.

Much of what is currently known about somatosensory-motor interactions has been derived from research combining neurophysiological techniques with behavioral measures of movement. In addition, behavioral and neurophysiology measurements in clinical populations affecting the basal ganglia, such as Parkinson's disease (PD), Huntington's disease (HD), and dystonia have been critical in advancing our understanding. Determining how somatosensory-motor interactions contribute to specific aspects of movements (e.g., initiation, inhibition, force production, timing) will help in understanding the role that these mechanisms contribute to sensorimotor pathophysiology of neurological disorders affecting the basal ganglia. Both animal and human research is critical to developing a thorough understanding of this topic. Furthermore, computational methods that consider the sensory-motor and connected biological system as a whole, including peripheral sensory receptor physiology, are integral to advancing knowledge on this topic.

In the Frontiers Research Topic, 12 papers (Table 1) have been published. Both from a methodological as well as from a theoretical view, many exciting contributions were published. The heterogeneity of the applied methods, e.g., movement analysis, diffusion tensor imaging (DTI), MEG, and non-invasive brain stimulation (NIBS), show how sophisticated the toolbox of the current neuroscience community is. In order to see how the frontier of the field has moved forward, one could ask oneself to which extent a contribution could have been realized 10 years ago. To answer this question, it is not only of relevance whether the method (hardware/software) was available 10 years ago but also whether the work builds on new ideas about the understanding of the role of the basal ganglia in health and disease.

**Table 1:** Summary of the published papers.

**References**	**Type**	**(Brief) Title**	**(Unique) Technique**	**Disease**
Beudel et al.	Perspective	Linking pathological oscillations to altered temporal processing in PD		PD
Cao et al.	Original	Dystonia cortico-subcortico coherence	LFP/MEG	Dystonia
Dubbioso et al.	Perspective	Fast intracortical sensorimotor integration	NIBS	PD
Feller et al.	Original	Sensory re-weighting for postural control in PD	Movement analysis	PD
Filip et al.	Original	Disruption of distinctive neural networks associated with ICD in PD	fMRI	PD
Filyushkina et al.	Original	Hyperactivity BGGL in PD during internally guided voluntary movements	fMRI	PD
Hirschmann et al.	Original	Longitudinal recordings reveal transient increase of alpha low beta in the STN	LFP/EEG	PD
Lee et al.	Original	Abnormal phase coupling in PD	EEG, NIBS	PD
Macerollo et al.	Original	Da modulation of sensory attenuation in PD	SSEP	PD
Martino et al.	Original	Motor timing in Tourette	DTI	Tourette
Milardi et al.	Review	CTX—BGGL—cerebellar circuitry		
Saxena et al.	Original	Modulation of GPi activity during hand movement	MER non-human primate	

*PD, Parkinson's Disease; ICD, impulse control disorder; BGGL, basal ganglia; STN, subthalamic nucleus; CTX, cortex; GPi, internal part of the globus pallidus; LFP, local field potentials; MEG, magnetoencephalography; fMRI, functional MRI; NIBS, non-invasive brain stimulation; SSEP, somatosensory evoked potentials; DTI, diffusion tensor imaging; MER, microelectrode recordings*.

One clear novelty in the field, that was not available 10 years ago, is the use of fully implantable DBS devices that not only can stimulate but can also record local field potentials (LFP's). The use of these devices as described by Hirschmann et al. gives an outstanding example of how these new technological advances can make invasive recordings from deep brain nuclei endlessly more easy, comfortable, and more comprehensive. The most important reason for this is that it is no longer necessary to perform LFP recordings in the intra- or immediate post-operative phase but that it is possible to conduct these recordings at any moment. Given the frailty of patients in the intra- or immediate post-operative after DBS implantation, conducting LFP research will be far less demanding from a patient perspective. One other crucial difference is that longitudinal recordings can be performed where changes in somatosensory-motor interactions can be studied over longer periods which might become a powerful tool to assess the efficacy of DBS or to develop biomakers, i.e., “physiomarkers” (Kühn and Volkmann, 2017) for adjusting DBS. The latter is called adaptive DBS (aDBS) and is currently a subject of intense inquiry (Habets et al., 2018).

Another development is the perception of the relevance of basal ganglia pathways beyond the direct, indirect and hyperdirect pathways as described by Milardi et al. Next to these “traditional” pathways, the traditional view that cerebellum and basal ganglia communicate via the cerebral cortex no longer holds and more relevant pathways exist between cerebellum and basal ganglia. These bilateral connections, i.e., cerebellum to basal ganglia and vice versa, have been revealed by state of art imaging methods like optogenetics showing short-latency activation (10 ms) of the basal ganglia after optogenetic stimulation of the cerebellum (Chen et al., 2014). These novel findings can be attributed to the sophistication of these new techniques over the last decade and would not have been possible 10 years ago. For this example, it is clear that new technologies clearly add to our understanding of the basal ganglia and restore concepts that have previously been discarded (Caligiore et al., 2017). Furthermore, the evidence for functional significance of the basal ganglia-cerebellar connections, and how these connections facilitate co-operation is growing and opens an entirely new perspective on the pathophysiology of movement disorders, like dystonia (Darby et al., 2019; Fung and Peall, 2019). Finally, these basal-ganglia cerebellar connections can now be functionally restored using DBS of the subthalamic nucleus (STN), leading to improved (sensori) motor learning (de Almeida Marcelino et al., 2019).

Next to the technological developments and new neuro-anatomical insights, a third recent and important development is the further understanding of the neural computations performed in the basal ganglia. In the contribution of Saxena et al. new physiological insights of the role of the basal ganglia in action selection have been provided. Traditionally, the way in which basal ganglia exerted their influence on each other or adjacent structures was thought to be based on the firing rate of individual neurons. This firing rate model was, however, challenged by the evidence that making a lesion can yield the same effect as stimulating the same structure using DBS and DBS of the same structure with the same stimulation characteristics can improve both hypokinetic (e.g., Parkinson's disease) and hyperkinetic (e.g., dystonia) movement disorders. In their paper, Saxena et al. showed that during movement planning and execution information encoding in the basal ganglia occurs in a highly localized manner via sudden emergence and suppression of oscillatory activities. More specifically, during movement oscillations in the gamma band (35–90 Hz) emerge and beta (13–30 Hz) oscillations decrease. This experimental evidence is in line with recent conceptions that neural activity in the basal ganglia is not strictly inhibitory or excitatory based on the firing rate but more on the firing pattern. In PD, strong evidence is available that shows abnormal oscillatory activity in this beta band that is correlated with the severity of motor symptoms (Ray et al., 2008). As such, the role of DBS might be more one of disruption of pathological oscillatory activity instead of simple inhibition or excitation (Chiken and Nambu, 2016).

This evident progress in both technology, basal ganglia connectivity and computational processing will find its way to clinical practice and will open up a plethora of research possibilities in the coming decade.

## Author Contributions

All authors have contributed to the editorial and critically reflected on the successive versions.

### Conflict of Interest

The authors declare that the research was conducted in the absence of any commercial or financial relationships that could be construed as a potential conflict of interest.
